# Commentary: Robots As Intentional Agents: Using Neuroscientific Methods to Make Robots Appear More Social

**DOI:** 10.3389/fpsyg.2018.01131

**Published:** 2018-07-20

**Authors:** Viktor Kewenig, Yuefang Zhou, Martin H. Fischer

**Affiliations:** Division of Cognitive Science, Potsdam Embodied Cognition Research Group, School of Psychology, University of Potsdam, Potsdam, Germany

**Keywords:** intentionality, social robots, verbal reports, humanoid, turing test

Robots can assist humans in a wide spectrum of domains (Tapus and Mataric, 2006; Cabibihan et al., 2013), including entertainment, teaching and health care. Since the need for assistance in these areas is steadily growing beyond what is currently possible with human workforce (Ward et al., 2011), research into social robotics is worthwhile. As Bartneck and Reichenbach (2005) pointed out, the general public can be quite skeptical with respect to the introduction of robot assistants in everyday life. For robots to be accepted as assistants, it is consequently important to understand the mechanisms that lead humans to conceive of robots as social companions.

The recently published paper by Wiese et al. (2017) is an appropriate response to the need for more research into this area and it comes at the right time. In it, Wiese et al. identify the higher-level activity of ascribing intentionality as the key mechanism for treating others as social companions. This much, it seems to us, must be correct. When we ascribe intentionality to someone, we give their actions meaning, we treat them as creatures with a mind that has the power “to be about, to represent, or to stand for, things, properties and states of affairs” (Jacob, 2014). Hence, ascribing intentionality to someone is a highly complex activity. It involves having *beliefs about someone else's beliefs*. In understanding the mechanism of ascribing intentionality, there are consequently *two* aspects that require consideration: the subject ascribing intentionality, and the object in question.

Wiese et al. consider both. First, they assume that mental states are visible in behavior. Accordingly, for the *object* to be ascribed intentionality, it should suffice for it to perform certain actions in certain ways. Their views seem to fit well with the recent argument on the relationship between movement features and perceived mental states (Becchio et al., 2017). Second, they think that in order to decide whether the *subject* is ascribing intentionality to the object, it is enough to look at neural activity in certain “social” areas of the subject's brain. Here we argue against Wiese et al.'s focus on a bottom-up approach, pointing out the importance of verbal reports in understanding the mechanism behind ascriptions of intentionality.

Having a belief means being in a semantically evaluable mental state. We conjecture that such activity is irreducible to neuroscience because of arguments based on Leibniz's gap as described in (Cummins, 2012, p 147):

“If we [examine] a machine whose structure makes it think, sense, and have perceptions […] we will only find parts that push one another, and we will never find anything to explain a perception.”

The point here is that thoughts cannot be observed or perceived solely by examining brain properties; beliefs are not reducible to brain states [e.g., (Richard, 1987) Kenny in Gregory (ed.)]. Given that we currently have no way of bridging Leibniz's gap between neural activity and intentional vocabulary, looking at a subject's brain might tell us *that* it ascribes intentionality to an object, but it won't help explaining *why* or *how*. In this sense, we argue, semantically contentful mental states are necessary for explaining the activity of ascribing intentionality. Verbal reports employing irreducible intentional vocabulary cannot simply be replaced by neuroscientific explanations.

The implications of this conclusion for the experimental design suggested by Wiese et al. are the following: in human-robot interaction, humans should be asked whether, for example, they think that the robot has certain beliefs and or goals in mind or whether its actions have a purpose. Including these kinds of verbal reports on top of neuroimaging would enable us to truly understand the mechanism behind ascribing intentionality through both a top-down and a bottom-up approach. Wiese et al. might respond that all they really need for their approach to work is “signposting”. Thus, they might claim that they only really want to see *that* a subject ascribes intentionality to an object, but not why and how it does so. We conjecture that this won't do. Their experimental design is thus rendered insensitive to possibly inhibiting factors, such as reasons for not adopting the intentional stance.

It is at least disputable whether or not robots will ever have a truly intentional mind (for example John Searle' Searle (1980) famous Chinese room argument). Until the question is settled, we cannot be sure that robots are *truly* intentional agents. As pointed out, Wiese et al. assume *pace* Searle that certain behavior is sufficient for the ascription of intentionality. But this seems too strong: even the Turing (1950) test suggests that we need *at leas*t verbal interaction to judge if someone is truly intentional. Granted, humans readily anthropomorphize and treat robots *as if* they were intentional agents. Neuroethicist Metzinger (2017) calls this “social hallucination,” yet this is clearly a far away from treating someone as a social companion. Remaining skepticism may likely inhibit adopting the intentional stance, even toward perfectly behaving robots; and thus keep people from treating social robots as *true* social companions.

Verbal reports present an easy way of detecting such inhibiting factors and of finding reasons why an agent might or might not treat a robot as social companion more generally. For example, in a human-robot interaction it is easy to ask whether the subject thinks that the object truly has a mind. Such information, conceptually, cannot be conveyed by neuroimaging. The experimental approach suggested by Wiese et al. is therefore insensitive to inhibiting factors like the one mentioned. Yet in trying to make robots appear more social, we clearly need to take them into account. The upshot must consequently be an experimental design that includes both neuroimaging and verbal reports.

We should mention that Wiese et al. do give some credit to what they call “subjective measures”, i.e., verbal reports. For example, they think that such measures are good in determining the likability of a robot. But this is too weak. Verbal reports are vital for questions regarding “mind perception,” i.e., whether or not we treat someone as an intentional agent.

## Author contributions

All authors listed have made a substantial, direct and intellectual contribution to the work, and approved it for publication.

### Conflict of interest statement

The authors declare that the research was conducted in the absence of any commercial or financial relationships that could be construed as a potential conflict of interest.
